# Image-based data on strain fields of microstructures with porosity defects

**DOI:** 10.1016/j.dib.2020.106627

**Published:** 2020-12-08

**Authors:** Pranav Khanolkar, Saurabh Basu, Christopher McComb

**Affiliations:** a Harold and Inge Marcus Department of Industrial Engineering, The Pennsylvania State University, University Park, PA, USA; b School of Engineering Design, Technology and Professional Programs, The Pennsylvania State University, University Park, PA, USA

**Keywords:** Microstructure, Strain fields, Images, Finite element analysis

## Abstract

The present article provides a compilation of microstructures and respective strain fields expressed by them during elastic loading. These microstructures were synthesized in Abaqus Standard software and their strain fields were modelled using Abaqus based static implicit analysis. The Python Development Environment (PDE) in Abaqus was used. These microstructures were subjected to uniform displacement boundary condition to obtain strain fields in the plane-strain mode. The purpose of the generating this data was to test the efficacy of convolutional neural networks (CNNs) in predicting strain fields. This raw data consisting of microstructure and their strain fields was converted to images using MATLAB as two dimensional arrays with each pixel denoting value to be used as input for training the CNN. This processed data in the form of images can be potentially used in deep learning or data science methodologies to perform finite element simulations.

## Specifications Table

**Table:** 

Subject	Engineering
Specific subject area	Training Data for Deep Learning Image Regression Computational Mechanics (FEA simulations)
Type of data	MATLAB Binary Data Compressed file (.mat)
How data were acquired	FEA simulations in Abaqus using the Roar Supercomputer
Data format	Raw image dataset
Parameters for data collection	Microstructure dimensions as per ASTM E8/E8M standards and strain fields analysis under specific boundary condition.
Description of data collection	Microstructure modelling and simulation were performed in Abaqus. Data from the simulations and microstructure model was converted to images (pixel-values) in MATLAB.
Data source location	Pennsylvania State University University Park, Pennsylvania, USA
Data accessibility	Repository name: Mendeley Data Data identification number: 10.17632/s4g76zd5ys.1 Direct URL to data: http://dx.doi.org/10.17632/s4g76zd5ys.1 Data identification number: 10.17632/jkkbg4d49g.1 Direct URL to data: http://dx.doi.org/10.17632/jkkbg4d49g.1 Data identification number: 10.17632/ns4bby8s6m.1 Direct URL to data: http://dx.doi.org/10.17632/ns4bby8s6m.1
Related research article	P. Khanolkar, C. McComb, S. Basu, Predicting elastic strain fields in defective microstructures using image colorization algorithms, Comput. Mater. Sci., vol. 186, doi: 10.1016/j.commatsci.2020.110068

## Value of the Data

• This data can be important in the field of computational mechanics as it provides a collection of images of microstructures with defects that have various geometric features.
• The collective image dataset can be useful as training data for researchers exploring machine learning methodologies.
• This data can also provide insights in conducting various image analyses including feature recognition and image colorization approaches for finite element analyses.

## 1 Data Description

**Fig. 1:**
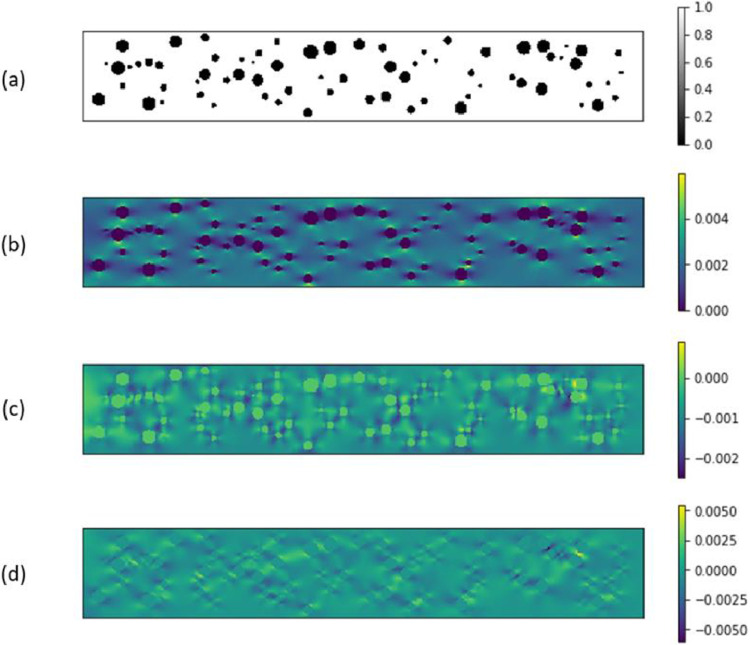
Example of microstructure with circular defects (a) Original microstructure layout; (b) Strain Field - ε_11_; (c) Strain Field - ε_22_; (d) Strain Field - ε_12_.

Each data file is a MATLAB Binary Data Compressed file (.mat) that contains images of microstructures and their respective strain fields represented as two-dimensional arrays. The original layouts of microstructures with defects have pixels values either 0 or 1 and are stored under ‘*defects*’ variable of each data file. The image representing arrays of three strain field components (ε_11_, ε_22_, and ε_12_), with pixel values denoting strain values, are stored under ‘*strain*’ variable of each data file. An example of a microstructure and its strain field components extracted from such data file is displayed in Fig. 1. The datasets for respective analyses described in the results section of research article [1, 2] have been stored in three folders as follows:

1. Training CNN and Prediction Time Comparison [3]:

This folder contains ‘data_1000_CNN.mat’ which comprises of image data of 1000 microstructures and corresponding strain fields.

2. Experimental Analyses_CNN vs FEA [4]:

This folder contains two sub-folders, each of them consisting image data of 10 microstructures and corresponding strain fields named ‘data_Exp1_radius_10.mat’ and ‘data_Exp2_no_of_pores_10.mat’ respectively.

3. Feature Learning Analysis of CNN [5]:

This folder contains six sub-folders each having two datasets (500 samples and 50 samples) each and have a naming convention as per following example:
*Sub-Folder ‘Microstructure Samples with Circular Defects’ contain files ‘data_circle_500_train.mat’ and ‘data_circle_50_test.mat’*

It is possible to open these .mat files using programming languages other than MATLAB. For instance, the files may be opened in the Octave programming language using syntax identical to that in MATLAB. They may also be opened in the Python programming language using the scipy.io.loadmat function in the SciPy library, in the Julia programming language using the matopen function from the MAT package, and in the R programming language using the readMat function from the R.matlab library.

## 2 Experimental Design, Materials and Methods

A code written in Python programming language was used to create the microstructure samples with porosity defects and perform finite element simulation in Abaqus Standard using Abaqus PDE (Python Development Environment). Each microstructure model was designed as per ASTM E8/E8M standards [6] with the material as aluminum (Al6061 – T6) and rectangular dimensions, 38.1 mm as length and 6 mm as breadth. In addition to that, Elasticity parameters corresponding to Al6061-T6 alloy were set as follows, i.e. Young's modulus as 68.9 GPa, Poisson's ratio as 0.35, along with density as 2700 Kg/m^3^. The position of defects in the microstructure were sampled from a uniform distribution within the boundaries of the rectangular specimen and were stored as raw data along with their geometric attributes such length, width, height, radius and aspect ratio, defined for respective defect shapes. The mesh and boundary condition of uniform uniaxial displacement of 0.1 mm in the plane-strain mode were specified for microstructure samples for finite element simulation to obtain strain fields. The results obtained from the simulation in the form of three strain fields (ε_11_, ε_22_, and ε_12_) were also stored as raw data in each of the three aforementioned cases, to be used for image conversion. The microstructure with defects of required shape, size and number was synthesized as per the three major analyses to be conducted to test the efficacy of machine learning framework used.

For the first and primary analysis of time comparison between the performance of convolutional neural network and finite element analysis software, 1000 samples of microstructures with 100 circular porosity defects having their radii uniformly distributed in the range 0.1 – 0.5 mm were synthesized.

In case of the second analysis that comprised of two experiments, two sets of 10 microstructures with circular porosity defects were generated for each experiment respectively. For the first experiment, the size of the pores in a microstructure was randomly varied from 0.01 mm to 0.5 mm in increments of 0.05 mm as (0.01, 0.05), (0.05, 0.1), (0.1, 0.15), till (0.45, 0.5), with each range distributed uniformly. The number of pores kept constant in these microstructures at 50. In the second experiment the size range of the pores was kept constant between 0.1 mm – 0.3 mm and the number of pores incremented from 20 till 200 in uniform increments of 20.

**Fig. 2:**
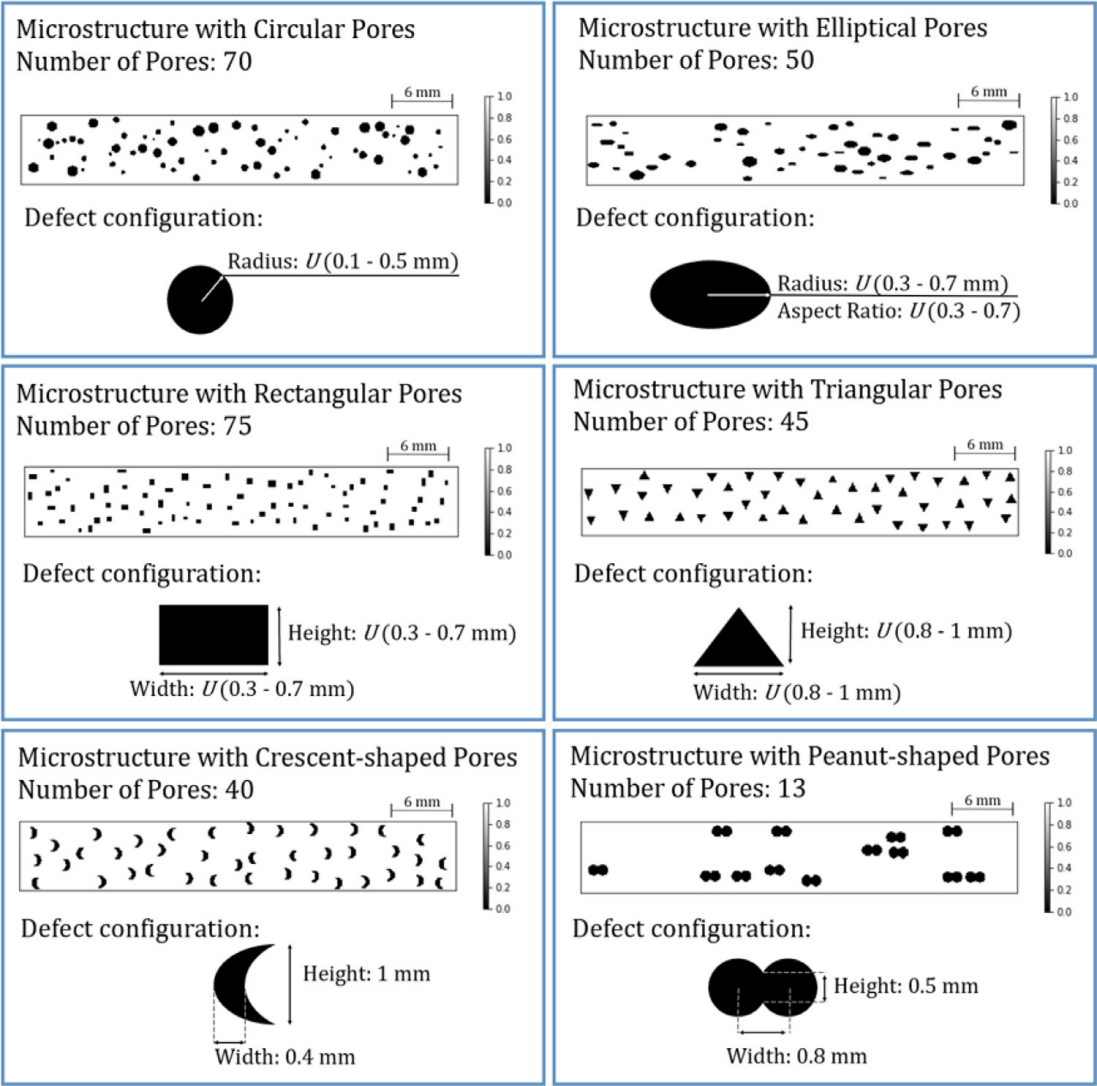
Microstructure samples having different shapes of porosity defects with area fraction 7% - 9% (modified from [1]).

For the feature recognition analysis, 500 training samples and 50 testing samples of each of microstructures with defect shapes mentioned in Fig. 1 were synthesized. The positions of the defect-shapes displayed in Fig. 2 were specified as follows: (1) Circle: Co-ordinates of the centre point; (2) Ellipse: Co-ordinates of the centre point; (3) Rectangle: Co-ordinates of bottom left corner; (4) Triangle: Co-ordinates of left most vertex; (5) Crescent: Co-ordinates of the centre points of the two arcs; (6) Peanut-shaped: Identical to the parameters defined for crescent shapes. The number of defects of any given shape in a specimens was determined by confining the defect area fraction within the range (7% – 9%). The number, size and shape parameters of the porosity defects (as depicted in Fig. 2) were specified in the code.

After obtaining the raw files describing the defects and strain fields, a code was developed in MATLAB script that provided the conversion of these raw data files into respective images. The code enabled discretization of the microstructures in the form of a square grid with a resolution of 0.06 mm/pixel that produced a shape of 101 × 636 pixels. This resolution was selected to preserve the major features of the microstructure with the minimum number of pixels. The area covered by the defects was assigned the pixel-value ‘0′ while the area covered by the material was assigned the pixel-value ‘1′. The defect zones were retrieved from the raw data and each shape was carefully assigned the ‘0′ value based on the shape and size parameters obtained from the raw data. Similarly, the raw data pertaining to the three strain fields (ε_11_, ε_22_, and ε_12_), was discretized on the 101 × 636 pixel^2^ square grid, with each pixel denoting the strain values at that particular position in the microstructure. Additionally, due to absence of material in the defect zones, the area covered by the defects was allotted ‘0′ pixel-value, based on the raw data consisting the defect locations and size-shape parameters. In such manner, each microstructure and their corresponding strain fields were converted to images each represented as a two-dimensional array with elements denoting pixel intensity of material, defect zone and strain value accordingly.

## Declaration of Competing Interest

The authors declare that they have no known competing financial interests or personal relationships which have, or could be perceived to have, influenced the work reported in this article.
